# Evaluating the Poppy Flower Program: A Person-Centered Communication Tool for Dementia Care

**DOI:** 10.1093/geroni/igaf122.2004

**Published:** 2025-12-31

**Authors:** Cassandra Dictus, Christy Knight, Eleanor McConnell

**Affiliations:** Duke University, Durham, North Carolina, United States; Durham VA Healthcare System, Durham, North Carolina, United States; Durham VA Healthcare System, Durham, North Carolina, United States

## Abstract

Hospitalization can be challenging for individuals with dementia, as interacting with unfamiliar environments and new caregivers often exacerbates cognitive and behavioral symptoms. These symptoms can contribute to challenges such as falls, elopement, and resistance to care. While staff may learn patient preferences over time, this knowledge is not always effectively elicited or shared. The Poppy Flower Program was introduced at the Durham VHA Medical Center to address this gap by improving access to patient-specific information, with support from the VHA’s Innovation Network’s Spark-Seed-Spread initiative. The Poppy Flower, a dry-erase board featuring a symbolic poppy of remembrance, was placed in patient rooms on four acute care medical-surgical units. Each petal included prompts (e.g., “I am upset by...” or “Important people and pets to me are...”) to facilitate communication and enhance person-centered care. This evaluation gathered staff and volunteer input to inform the spread phase of the program by describing the frequency of Poppy Flower use, how staff use the tool, and the perspectives of frontline staff and leadership on the program’s value and effectiveness. Preliminary findings suggest that while some staff view the Poppy Flower as a useful tool for communication, its use has been inconsistent. Barriers include the placement of the Poppy in the room, workflow integration challenges, and a task-driven care culture where staff were less accustomed to engaging with patient background information. Identifying barriers and facilitators will help determine whether to continue, modify, or expand the program to better support staff in providing high-quality dementia care.

